# The Library

**Published:** 1912-08-24

**Authors:** F. G. Haley

**Affiliations:** National Liberal Club, Whitehall Place, S.W.


### The Editor's Letter-Box.

### "The Library."

*To the Editor of* The Hospital.

Sir,—Your correspondent, in his interesting article, "The Library," in the series of "Relaxations and Hobbies," is in error in saying (on p. 517 of The Hospital of August 17) that the minimum subscription to the London Library is £10. The subscription is £3 per annum.—Yours faithfully,

F. G. Haley.

National Liberal Club, Whitehall Place, S.W.
August 19.

